# Nutritional Biomarkers, Gene-Diet Interaction, and Risk Factors for Type 2 Diabetes

**DOI:** 10.1155/2016/8610501

**Published:** 2016-12-25

**Authors:** Ju-Sheng Zheng, Kaijun Niu, Simone Jacobs, Hassan Dashti, Tao Huang

**Affiliations:** ^1^MRC Epidemiology Unit, University of Cambridge, Cambridge, UK; ^2^Nutritional Epidemiology Institute, Tianjin Medical University, Tianjin, China; ^3^Institute of Public Health, University of Heidelberg, Heidelberg, Germany; ^4^Center for Human Genetic Research, Massachusetts General Hospital, Harvard Medical School, Boston, MA, USA; ^5^Saw Swee Hock School of Public Health, National University of Singapore, Singapore

Prevalence of type 2 diabetes (T2D) has been increasing globally, gaining high public health priority [1]. T2D is a complex disease, resulting from an interplay between lifestyle and genetic factors. Evidence from several randomized controlled trials suggests that physical activity and dietary intervention could prevent or delay progression of T2D [2–4]. In addition, genome-wide association studies have identified hundreds of genetic variants associated with T2D [5]. In the present special issue, each paper investigated different aspects of T2D prevention strategy, ranging from physical activity and diet/nutritional biomarker to gene-epigenetic mechanisms, in animal models and human studies.

J. Qi et al. revealed that, in rats with insulin resistance induced by a high-fat diet, swimming exercise could improve insulin sensitivity, potentially via a reduction of TRIM72 expression, and upregulation of PI3-K/AKT signaling pathway, including IRS-1, p-AKT^Ser473^, and AKT expression. The PI3-K/AKT signaling pathway is critically important for the regulation of insulin sensitivity and other biological processes related to aging [6, 7]. This study further showcased the intricate mechanism linking physical activity (swimming in this study) to insulin resistance. With similar design in a high-fat induced nonalcoholic fatty liver disease rodent model, Y. Xie et al. found that total alkaloid from Nelumbinis Plumula (NPA) could reduce insulin resistance via mechanism of restoring IRS1 and suppressing the JNK phosphorylation. Indeed, natural products derived from food resources have been proved to be promising natural remedies for T2D prevention and treatment [8].

Objectively measured nutritional biomarker may overcome recall bias and measurement error resulting from traditional dietary assessment tools, such as food frequency questionnaires. Emerging metabolomics studies are revealing more and more promising nutritional biomarkers and linking them to health outcomes. One of the examples is Branched-Chain Amino Acid (BCAA). In this issue, X. Zhao et al. systematically reviewed the current evidence on the relationship between BCAA and insulin resistance and concluded that circulating BCAAs (including valine, leucine, and isoleucine) are positively associated with insulin resistance and are useful biomarkers to predict future T2D cases. In a recent Mendelian randomization analysis, researchers identified a causal association of high circulating BCAAs with T2D risk and proved the causal role of BCAA metabolism in the T2D etiology [9]. Thus, use of nutritional biomarkers has obtained increasing popularity given their nature of objective measurement and potential usage to test causality in observational studies.

Lastly, L. Yang et al. updated the association of genetic variations in* LEPR* gene with T2D risk with a meta-analysis, providing robust evidence for the association between rs1137101 at* LEPR* and T2D risk. In addition, T. Matsha et al. demonstrated the role of DNA methylation-genetic variations in screen-detected diabetes or known diabetes on treatment in an African population. All the above efforts add to the current knowledge on the role of genetic and epigenetic modulation on T2D etiology [10].

Taken together, articles in this special issue highlight several important fields in the prevention and treatment of T2D, focusing on the important role of diet and physical activity, incorporating natural remedies from food resources and new omics (metabolomics and genomics) strategies. The issue also suggests the importance of integrative strategies for the prevention and treatment of T2D in future research.

*Ju-Sheng Zheng* *Kaijun Niu* *Simone Jacobs* *Hassan Dashti* *Tao Huang*
